# Lessons from the health policies for children during the pandemic in Japan

**DOI:** 10.3389/fpubh.2022.1015955

**Published:** 2022-10-06

**Authors:** Naohisa Shobako

**Affiliations:** Division of Food Science and Biotechnology, Graduate School of Agriculture, Kyoto University, Uji, Japan

**Keywords:** food education, stay at home, masks, COVID-19, pandemic, health policy

## Abstract

It is everyone's desire to seek the sound growth of children through food education and there is a critical need for fostering an environment for this purpose. Health policies are important for this support. To the present, the Japanese society has been greatly disrupted by COVID-19 pandemic. “Stay at home”, “mokusyoku (silent eating)”, and mask wearing were encouraged in nationwide campaigns as public health measures to combat COVID-19. There are some papers reporting negative effects of “stay at home” and lockdowns such as weight gain, decrease in physical activities and change in eating habits. In Japan, while benefits and advantages of food education during mealtime were previously well studied, the “mokusyoku” rule may directly run counter to this food education. Moreover, there are several reports showing that nutrients might contribute to prevention of infectious diseases. Japanese children were also encouraged to wear masks all day long. The results of the clinical research, especially randomized control trials, show limited protective effect of masks. On the other hand, negative outcomes of the masks were reported in various scenes. This review focuses on these topics and arousing reconsideration for a better environment for children.

## Introduction

Eating, learning, and playful behaviors are essential for healthy development of children. Societies around the world, including Japan, have been severely affected by the COVID-19 pandemic. It was well announced that Japan had controlled the infection successfully without draconian lockdowns or other harsh restrictions which unduly limit private rights of citizens.

**Figure 1:**
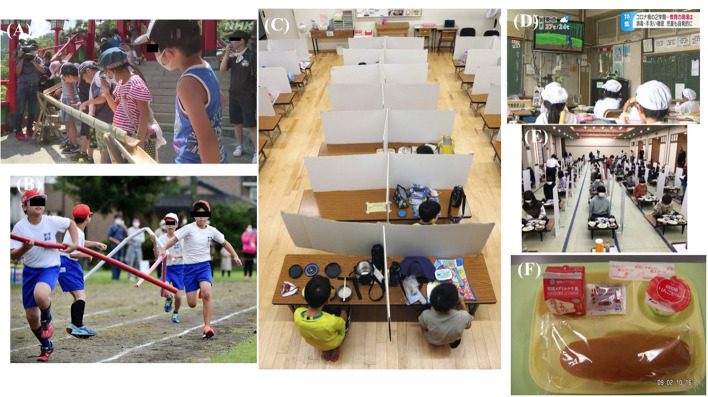
Example of “Infectious disease counter measures” for children taken for Japanese Children. **(A)** Children watching “somen (Japanese traditional noodle) flow” with their mask on in silence in summer. Under ordinary circumstances, children eat somen noodles flowing in front of them. **(B)** Special care is taken to keep “Physical distance” in a relay race of a school sport festival. The length of the baton was 2 meters. **(C)** Children are taking lunch following “mokusyoku rule”. **(D)** Children are watching TV program during lunch time to follow mokusyoku rurle. **(E)** “The New way of lifestyle” adopted in a school excursion. Children must follow “mokusyoku rule”. **(F)** Example of “Simple school lunch”. These pictures were taken from the following websites: **(A)** “Watch over them without eating. Somen flow in Tsuwano city preventing COVID-19 infection. 8/9/2020” Japan Broadcasting Corporation https://www3.nhk.or.jp/news/html/20200809/k10012560081000.html. **(B)** “2 meter baton, shouts on paper, broadcast on TV sports festival under COVID-19 pandemic. 9/27/2020” Mainichi Shimbun https://mainichi.jp/articles/20200927/k00/00m/040/122000c. **(C)** “Infection for children is increasing in COVID-19 seventh wave. What are the characteristics of the symptoms? What measures are needed for the new semester? 8/26/2022” Tokyo shimbun https://sukusuku.tokyo-np.co.jp/education/59471/. **(D)** “A strategy to follow mokusyoku rule by showing animated films during lunch time. In the second semester of the COVID-19 epidemic at an elementary school, the educational scene is undergoing a trial-and-error process for infection prevention measures. Nagano city. 9/1/2022” Shin-etsu broadcasting https://newsdig.tbs.co.jp/articles/sbc/140832?display=1. **(E)** “Dinner with mokusyoku rule and infection control measures 11/25/2021” Official Blog of Onohara east primary school http://www.kamisu.ed.jp/onoharanishi/19314.html. **(F)** Fuji News Network “Zero side dishes painful choice “simple school lunch” to prevent COVID-19 infection, 1.2 times the amount of hot dog buns, and concerns about nutritional deficiencies. 9/8/2021” https://www.fnn.jp/articles/-/235832. For privacy, part of the face is hidden. All sites were accessed at September 19th, 2022.

However, the environment in which children eat, learn and play has greatly been affected by health policies in Japan. While no proven effect of school closures against spread of COVID-19 has been reported (1), a few schools are still taking temporary closure measures (2). Though most schools were opened, children were strongly encouraged to follow the “mokusyoku” rule of eating lunch silently during the lunch time (3). Although the Ministry of Education, Culture, Sports, Science and Technology (MECSST) has modified the guideline of mask wearing in schools to clarify that mask wearing is not necessary in physical education classes as long as the social distance (1–2 m) is maintained, the ministry still recommended to continue the mask wearing rule in schools (4). Typical events related to COVID-19 and children are summarized in Table 1. As shown, novel Coronavirus Response Headquarters present the basic policies for COVID-19 prevention, and each ministry announces relevant basic policies (5). MECSST presents guidelines describing the basic policy of countermeasures that the schools should take. Since most of them are only written recommendations, each municipality's board of education and school finally decide what kind of countermeasures to request for children. So, children were forced to comply with many kinds of measures. Examples of such measures are shown in Figure 1. As shown in the figure, children were forced to follow “The New Lifestyle (New Normal Lifestyle)” in the name of public health.

**Table 1:** Typical events about history of COVID-19 pandemic in Japan.

**Year**	**Month**	**Typical event**	**Topics with stay at home, mokusyoku, and masks**
2020	January–March	- A man who stayed in Wuhan tested positive for COVID-19 (first case in Japan). - COVID-19 cases were confirmed among passengers aboard the Diamond Princess, which called at the port of Yokohama. - School reopening guidelines in response to COVID-19 (school reopening guidelines) was announced.	- Prime Minister Shinzo Abe requested the simultaneous closure of all elementary and junior high schools nationwide. - The Governor of Tokyo requested voluntary abstinence from going out.
	April–June	- State of emergency declaration (~25th May). - New Normal Lifestyle was presented by MHLW. - Hygiene Management Manual at Schools (hygiene manual) ver.1 was announced. - Simple school lunch was described as thoughtful for areas where infection is judged to be spreading.	- School reopening guidelines was updated. “In school education activities, please wear a mask regularly.” was added. It was also stated that masks must be worn in situations where close-range conversations or vocalizations are required. - The Governor of Tokyo announced “Stay home week.”
	July–September	- The “Go to travel campaign” started.	- The Governor of Tokyo required voluntary abstinence from home coming visit during summer vacation.
	October–December	- Interruption of the “Go to travel campaign.” - The hygiene manual was updated to ver.5. Especially for elementary and junior high schools, it is clearly stated that temporary closures for the entire region should basically be avoided.	- For junior and senior high school students, it was stated that, depending on the infection situation, activities that pose a high risk of infection without wearing a mask should be avoided in the hygiene manual (ver.5). - The Governor of Tokyo called for “Stay at home” during the New Year holidays. - Subcommittee on Novel Coronavirus Disease Control, proposed “mask dinner*”. Several local government are still requesting this practice.
2021	January–March	- State of emergency declaration (8th January−21th March). - COVID-19 vaccination was started.	- The Governor of Tokyo announced “Stay home thoroughly.”
	April–June	- Pre-emergency measures (5th March−30th September). - State of emergency declaration (25th April−20th June).	- About 7,000 children were reported to be voluntarily missing school due to fear of infection. - A child died after an endurance run in school with a mask was placed on the chin. - The Governor of Tokyo announced “Stay home week”.
	July–September	- The 2020 Summer Olympics was held in Tokyo.	- The Governor of Tokyo called for “stay home” during summer vacation. - There were requests for the extension of summer vacation due to fear of infection, and some schools responded.
	October–December	- COVID-19 vaccine booster shots were started.	- School closures, class closures were undertaken in Sapporo. - Novel Coronavirus response headquarter (belongs to Japanese government) announced basic policy against for COVID-19 (basic policy) and “Mokusyoku” was set as the basis.
2022	January–March	- Pre-emergency measures (7th January−21th March). - Vaccination for children (aged 5–11 years) started.	- The Governor of Tokyo called for “stay at home” hence the chairman of subcommittee on Novel Coronavirus Disease Control commented that it is not always necessary.
	April–June	- Latest guidelines for the school hygiene manual were updated (ver.8), a description about excessive sterilization was added. - Fourth shot started.	
	July–September	- Expanded the fourth vaccination target (e.g., healthcare workers). - Japan recorded the world's highest number of new COVID-19 infections.	- The MHLW homepage was updated about COVID-19 prevention. “Mokusyoku” is still the basis of the prevention response. In response to this, some boards of education and schools are encouraged to follow “mokusyoku” rule. On the other hand, some school boards have declared that they do not strongly recommend that.

Topics were taken from the homepage of the Japanese public broadcaster (https://www3.nhk.or.jp/news/special/coronavirus/chronology/), Tokyo metropolitan government (https://www.metro.tokyo.lg.jp/tosei/governor/governor/), the ministry of education, culture, sports, science and technology (https://www.mext.go.jp/a_menu/coronavirus/index_00012.html), the ministry of Health, Labor and Welfare (https://corona.go.jp/emergency/).

^*^A series of manners in which mask is removed with strings over the ears only when food is placed in the mouth and replaced immediately while chewing. This is sometimes seen in Japanese TV programs. However, it is now generally understood, as recommended by Kanagawa prefecture, that following the “mokusyoku” rule is more suitable during eating and wearing masks only when conversing (https://www.pref.kanagawa.jp/docs/r5k/mask_nisho.html).

An object of this paper is to summarize the findings about the result of health policies taken in Japan and looking back the challenges of science-based policy making.

## “Stay at home” campaign

Draconian lockdowns were enforced in many regions in the world such as China and Europe to prevent the wide-spread of COVID-19. “Stay at home” campaign was also strongly promoted in Japan even though Japan did not adopt any lockdown with severe restrictions. During the campaign, while adults were not subject to commuting restrictions, children were forced to stay at home due to the long-term school closures. Some negative effects of the lockdowns and “Stay at home” campaign were previously reported. In Japan, during the state of emergency, schools were closed and a “stay-at home order” was issued. As a consequence, it was reported that children had higher body fat percentage, shorter single leg standing time, and a larger number of falls per month compared with children before the pandemic (6). Abe also reported that fundamental movement skills, especially for object control skills were impeded during this pandemic (7). In low-income households, children's consumption of sweets, soft drinks, and ready-to-eat foods was increased (8). Horikawa et al. also reported children eating a balanced diet of meat or fish and vegetables at least twice a day decreased during the period (9). Horikawa's research also revealed the importance for the support of low-income households. Changes in eating habit was also observed in Kosaka's study and interfaced with feeling enervation and mental stress (10). Longer periods for video games were also reported in this study. Ueda et al. reported about half of children, participated in the research, experienced sleep pattern change during the period and its change was predicted by a high level of depression (11). In Nakachi et al. study, change tendency in sleep pattern was also observed in junior and high school students (12). Psychological problems were observed in lower grade of elementary school; they easily cried and complained, were unable to keep calm, and were dependent on parents and family members.

Similar results were reported outside Japan, where stronger behavioral restrictions have been imposed. For example, in China, significant increase in total food intake, especially snacks and drinks, and decrease in physical activities were reported (13). Negative effects of weight gain were also reported in research in U.S. (14, 15). Changes in eating habits and lifestyle during lockdowns were also reported (16, 17). Increased risk in type 2 diabetes was also reported (18). The restrictive lifestyle and weight gain due to the lockdown are considered be the causes of the increased risk in type 2 diabetes. The survey conducted on children also showed a significant increase in the consumption of potato chips, red meat, and sugary drinks, and a significant decrease in time spent in sports (19). The studies presented in this section are summarized in Table 2A.

**Table 2:** The typical studies about stay at home (A), mokusyoku and food education (B), and RCTs about mask-wearing (C).

**(A) Studies about “stay at home”**			
**References**	**Country**	**Participants (year old)**	**Overview of the results and notable points**
Ito et al. (6)	Japan	6–7	Children after the state of emergency had significantly higher body fat percentages, shorter single-leg standing times, and a larger number of falls per month than before.
Abe et al. (7)	Japan	3–5	Consumption of snacks, juice, instant foods, and canned food during the state of emergency, was higher in the low income group than in the high income group.
Sakamoto et al. (8)	Japan	3–5	Consumption of snacks, juice, instant foods and canned food during the state of emergency, was higher in low income group rather than high income group.
Horikawa et al. (9)	Japan	10–14	“Well-balanced dietary intake” was lower in all households during the state of emergency compared with before. The authors discussed that schoolchildren's meal quality worsened during the state of emergency, especially in low-income households, because school lunches were not provided.
Kosaka (10)	Japan	First–fifth grade (6–11)	There were significant differences in “irregular sleep,” “disordered eating habits,” and “increased use of games and smartphones”, during school closure.
Ueda et al. (11)	Japan	8–17	During the COVID-19 stay-at-home period, 46.5% of participants experienced changes in sleep patterns. These changes were associated with decreased QOL as well as internalized symptoms. The decreased QOL of children with altered sleep patterns was predicted by a high level of depression.
Nakachi et al. (12)	Japan	6–18	Children in the lower grade elementary school group easily cried and complained during quarantine (12.4%) and it was more difficult to keep calm compared to those in the other groups. Changes in sleep patterns were more prevalent in junior and senior high school students.
Zhu et al. (13)	China	16–70	There was a significant increase in total food intake especially in snacks and soft drinks under “stay at home regulation”. A significant decrease in physical activity was also observed. The rate of weight gain in the total population was 30.6%. The main factors contributing to weight gain were increased food intake and reduced physical activity.
Zachary et al. (15)	U.S.	Over 18	22% of adults report having gained weight during the COVID-19 pandemic. Lack of sleep, decreased physical activity, snacking after dinner, eating in response to stress, and eating because of the appearance and smell of food are reported as behaviors linked to weight gain.
di Renzo et al. (16)	Italy	Over 12	The perception of weight gain was observed in 48.6% of the population during lockdown. Consumption of homemade sweets and pizza was increased notably. But some good trends were also observed; 15% of respondents turned to farmers or organic purchasing groups for fruit and vegetables, especially in the North and Center of Italy, where BMI values were lower. Younger people (aged 18–30) tended to consume a more Mediterranean diet.
Ghosal et al. (18)	India	Not clearly described	There was a trend toward weight gain (0.1–5.0 kg) seen in 40% of the cohort, with 16% of the population experiencing a 2.1–5.0 kg weight increase.
Pietrobeli et al. (19)	Italy	6–18 (with obesity)	Consumption of potato chips, red meat, and sugary drinks increased significantly during the lockdown. Time spent on sports activities was significantly decreased and sleep time was significantly increased. Screen time was also significantly increased.
**(B) Papers reporting “mokusyoku”/studies about “food education”**
**References**	**Topic**	**Participants (years old)**	**Overview of the results and notable points/how is described about “mokusyoku”**
Noi et al. (3)	Mokusyoku		Students are forced to live in cramped and suffocating conditions, wear masks, and follow “mokusyoku rules,” and school events are canceled or curtailed. The author expressed concern about the effect on childrens' minds and bodies.
Okuyama and Seto (20)	Mokusyoku	Adults wit/without children	The survey revealed that parents with children in elementary school are concerned about the negative impact of “mokusyoku”.
Kishida and Kamimura (23)	Food education	Fifth–sixth grade (10–12)	The conversation-positive group gained higher scores in numerous items; good appetite, awakening feeling well, not feeling fatigue, sleeping well at night, not readily catching cold. Positive effects, including improved eating habits and reducing soft drink consumption were also observed.
Esaki (24)	Food education	Junior high school student (12–15)	The author reported that number of menus and people sharing meals, and helpful behavior are involved in improving quality of life (QOL).
Tominaga et al. (25)	Food education	Junior, high, university school student	Eating with having fun is associated with university personality inventory score, representing mental health.
Eto et al. (26)	Food education	5th and 8th grade student	Attitudes toward eating (communication) was associated with QOL especially in 8th grade (junior high school) students.
Nakamura et al. (27)	Food education	30–59	Higher household income and education levels were significantly associated with higher rates of eating vegetables, using the information on nutrition labels, and conversing with family or friends during meals. Higher household incomes were also significantly associated with lower frequencies of family breakfasts in men and a lower frequency of family dinners.
Barnes et al. (28) and Gest et al. (29)	Food education	3–4	Children more prone to decontextualized talk, positing a key role for language learning during mealtime rather than free play and reading time.
**(C) Overview of typical RCT trials about masks**			
**References**	**Participants**	**Group (** ***n*** **)**			**Intervention period**	**Overview of the results and notable points**
		**1**	**2**	**3**		
Cowling et al. (55)	Households	Control (*n* = 74 index cases, 213 contacts)	Mask (*n* = 22 index cases, 65 contacts)	Hand hygiene (*n* = 32 index cases, 92 contacts)	9 days	The secondary attack ratios did not significantly differ across the intervention arms.
Cowling et al. (57)	Households	Control (*n* = 91 index cases, 279 contacts)	Hand hygiene (*n* = 85 index cases, 257 contacts)	Mask + hand hygiene (*n* = 83 index cases, 258 contacts)	1 week	The differences from the control group were not significant.
Maclntyre et al. (59)	Households	Control (100)	Surgical mask (94)	P2 mask (92)	1 week	No significant difference in ILI was observed in each group, even in the control vs. all types of masks.
Jacobs et al. (62)	Healthcare workers	Control (17)	Surgical mask (15)		11 weeks	Benefits in the prevention for cold symptoms were not observed. Days with headache was significantly longer in the mask group.
Aiello et al. (52)	Students living in university residence halls	Control (552)	Mask (378)	Mask + hand hygiene (367)	6 weeks	No significant difference was observed in group 2. Group 3 showed significant suppression of ILI at week 4–6. The Cochrane review excluded from the meta-analysis because of insufficient randomization. This review also pointed out unclearness of the adjustments and exclusions at the baseline.
Larson et al. (53)	Households	Control (*n* = 148 households, total 904)	Hand hygiene (*n* = 148 house holds, total 946)	Mask + hand hygiene (*n* = 147 households, total 938)	19 month	There were no significant differences in rates of infection by intervention group in the multivariate analyses. The Cochrane view pointed out that randomization and reasons for dropout were not clearly described. It was also suggested that differentials in cluster characteristics across arms point to randomization not having worked.
Canini et al. (54)	Households	Control (*n* = 53 index cases, 158 contacts)	Mask (*n* = 52 index cases, 148 contacts)		1 week	No trend suggesting effectiveness of masks was confirmed. Pain was reported significantly more in children than adults in the mask group.
Simmerman et al. (56)	Households	Control (*n* = 119 index cases, with 302 members)	Hand hygiene (*n* = 119 index cases, with 292 members)	Mask + hand hygiene (*n* = 110 index cases, with 291 members)	3 weeks	Influenza transmission was not reduced by interventions. ILI in treatment group 3 was significantly higher than the control group (OR = 2.15; 95% CI: 1.27–3.26).
Aiello et al. (51)	Students living in university residence halls	Control (370)	Mask (392)	Mask + hand hygiene (349)	6 weeks	Both intervention groups compared to the control showed cumulative reductions in influenza rates over the study period, although the results did not reach statistical significance. A significant reduction in ILI was not observed in group 2 while group 3 showed in week 3–6.
Suess et al. (50)	Households	Control (82)	Mask (69)	Mask + hand hygiene (67)	8 days	There was no statistically significant effect of the interventions on secondary infections.
Barasheed et al. (63)	Hajj pilgrimage	Control (89)	Mask (75)		5 days	There was no significant difference in laboratory-confirmed illnesses, while ILI was significantly lower in the mask group (*p* = 0.04).
Maclntyre et al. (60)	Healthcare workers	Control (458)	Cloth mask (569)	Surgical mask (580)	4 weeks	The risk rate of the medical mask group was not significantly different from control group, but higher in cloth mask group (ILI).
Maclntyre et al. (59)	Households	Control (*n* = 122 index cases, 295 contacts)	Mask (*n* = 123 index cases, 302 contacts)		1 week	No statistically significant difference was observed.
Alfelali et al. (58)	Hajj pilgrimage	Control (3139)	Mask (3199)		4 days	No significant difference was observed in laboratory- and clinically-confirmed infections.
Abaluck et al. (71)	Villagers	Control (*n* = 163,861)	Mask (surgical and cloth; *n* = 178,322)		8 weeks	COVID symptoms were significantly decreased in the treatment group. The significant effectiveness of surgical mask was observed only in the ≧50 years-old subgroup. A commentary pointing out unignorable biases was provided by Chikina et al.
Bundgaard et al. (49)	Community	Control (*n* = 2,740)	Mask (*n* = 2,392)		4 weeks	There was no significant difference in COVID-19 infection between two groups. A total of 52 participants in mask group and 39 control participants reported COVID-19 in their household.

ILI, Influenza like illness.

## “Mokusyoku rule” and food education

The “Mokusyoku rule” is prohibition of conversation during meals in schools, working spaces, and restaurants (3, 20). The novel Coronavirus Response Headquarters announced “mokusyoku” as a basic policy for COVID-19 prevention (5), and the Japanese government and industry groups are promoting this health policy by spreading awareness (21). As described in Section Introduction, the final decision relies on each municipality; some local governments relaxed the “mokusyoku rule” in schools, while others continue to instruct children to follow. In order to ensure the rule implementation, each school is trying to search for the best way, such as using partition or TV animation even in 2022 (Figures 1C,D). In the early stage of the pandemic, droplet infection was thought highly threatening, and measures aimed at an assumed droplet pathogen were over-emphasized (22). The mokusyoku rule was thought to be a remnant of that time, the same as surrounding individuals with panels (Figures 1C,E). There are no reports on the benefits of the “mokusyoku rule,” and several Japanese articles have expressed worry about or negatively commented on its impact on children (20).

The importance of conversations during meals for children has been well studied in Japan. Kishida and Kamimura reported conversation positive group (group with frequent conversation) gained higher scores for good appetite, not feeling fatigue, sleeping well, and not readily catching cold (23). There are also reports of positive effects on eating habits and reducing soft drink consumption. Esaki reported frequent conversation during meals has positive relation with meal-related quality of life (QOL) (24). Previous studies also showed that Japanese children who had conversations during meals had better dietary attitudes, eating behavior and mental QOLs (25–27). Surveys outside Japan have also reported that conversations during meals in pre-kindergarten are effective for vocabulary acquisition because out-of-context conversations occur uniquely (28, 29). Although some papers suggest that people have indeed contracted COVID while eating in restaurants, such risk can be minimized by ventilation which is an important factor in preventing COVID (30, 31). As described above, conversations during meals are important for children to foster healthy minds and eating habits. Thus, it will be necessary to reconsider the “Mokusyoku rule” that would adversely affect physical and mental development and abolish the rule by taking measures such as sufficient ventilation. Articles describing mokusyoku and food education are summarized in Table 2B.

Some schools introduced “simple school lunch” with insufficient nutrition for fear of contact infection at the time of serving the meal (Figure 1F). This has been described as thoughtful for areas where the infection is judged to be spreading, as per the first version of hygiene management manual at schools to the latest version, established by MECSST (4, 32). Tanaka et al.'s survey showed simple school lunch was served in a certain number (55/205 schools for 10–40 days) of schools (33). The relationship between nutrients and infectious disease has been well studied (34). Vitamin D (VD) is probably the most well studied nutrient which has been reported to have a protective effect against COVID-19 infection (35). It is reported that serum 25-hydroxyvitamin D [25(OH)D] of <20 ng/mL is one of the risk factors of deficiency and according to a survey in South Korea, serum 25-hydroxyvitamin D [25(OH)D] of about the half of the 6–12 years old children was 20 ng/mL or less (36, 37). Not only from the diet, but also exposure to sunlight is important for vitamin D synthesis. During the pandemic, children's serum 25(OD)D concentration was significantly decreased. It was discussed that school closures and lockdowns were associated with this decrease (38). In the Turkey observational study for children under 18 years old, VD deficiency was significantly high in the COVID-19 patient group compared with the control group (39). Vitamin C (VC) and omega-3 fatty acids were also considered to prevent or reduce COVID-19 infection by cytokine modulation such as IL6, TNFα, and IL1β reduction and IL10 upregulation (40). Although some sufficient clinical observations have been reported, there are few data supporting active intervention especially for children (41). The situation of Vitamin E, considered as a natural killer cell and a T cell activator, was similar to VC (42). For children, there are a few RCTs showing positive effects of Vitamin A for preventing respiratory infections. However, the results of meta-analyses did not support active intervention (43). Zinc is well known for its important role for the development and maintenance of immune and other cells (44). Previous studies revealed that low zinc status is a risk factor of pneumonia infection for children (45). RCT studies for children also support the importance of Zinc (46). It was also reported that low selenium status is associated with COVID infection (47). Simple school lunch might be leading to opportunity loss of taking these nutrients.

Horikawa et al. discussed that school lunches play an important role in continuing well-balanced eating habits (9). Detailed research about the nutrition of simple school lunches and their effect has not been performed, but Kojima reported that it might not provide necessary nutrients compared to regular school lunch, while there was an apparent effort in the areas where a state of emergency was declared for a long time (48).

## Mask rules (mandate): Review of its effectiveness

Wearing surgical masks was strongly recommended in Japan even for children in school, on the way to and from school, and even in the house (so called “family mask” in Japanese; Yamanashi Center for Infectious Disease Control and Prevention). Randomized control trials (RCTs) with appropriate sample sizes have reported the limited effectiveness of surgical masks for infectious diseases, COVID-19 and influenza (49–63). Especially, RCT demonstrated by Simmerman et al. showed significantly opposite effect in Influenza like illness (56) and demonstrated by Jacobs showed only significantly prolonged the duration of the headache (62) (Table 2C).

Significant effectiveness of masks for COVID-19 prevention was reported in numerous observational studies (64–70) and “Bangladesh study” (71). Regarding the “Bangladesh study”, there are some points to be noted in the interpretation of the results. First, the total sample size was too large to conduct proper evaluation (*N* = 342,183), and subgroup analysis revealed that no significant prevalence intervention ratio was observed in the age 50 subgroup for surgical mask. Second, increase of the physical distance was observed in mask group but not in the control group. Third, monetary reward was provided for participants. Chikina et al. recently reported the re-analysis results and pointed out potential biases that cannot be ignored (72). In support of this view, based on the results of meta-analysis of RCTs, the universal mask policy especially in community settings is not strongly recommended (73–75). While many observational studies reported on the effectiveness of face mask, Davies et al. pointed out that most of them were based on self-reporting, and <0.2% of studies studied the behavior in question objectively (76). Particularly, the frequency of hand washing tends to vary greatly between the actual and self-reported values, which might be the reason for overestimating mask-wearing effectiveness. Frequency of self-report mask use was also reported to differ from actual. Thus, we should carefully consider this when determining the effectiveness of personal protective equipment by observational studies. Given the sample size and results of RCTs, it may be necessary to reconsider overestimation of mask effectiveness for scientific integrity.

Effectiveness of the mask mandate should have been reconsidered as well. The survey in Europe and Texas state revealed that the mas mandate has no effect against COVID-19 infection, hospitalization, and mortality (77, 78). In Kansas, counties with mask mandate had significantly higher case fatality rates than counties without mask mandate, with a risk ratio of 1.85 for COVID-19-related deaths. The mechanism of this adverse effect is propounded as “Foegen effect” (79). This effect was supported by *in vitro* examinations. In the manikin model, favorable results of viral titer or viral RNA detection were observed when the receiver was not wearing a mask (80). It will be necessary to consider what happens if the simulation is continued for more than 20 min, extended from the experimented time. This paper also points out an important issue. The droplets captured by the mask might be transformed into aerosols and were floated in a chamber. Penetration and secondary atomization of droplets impacted on the surgical masks were also well studied (81, 82). People wear masks for a long period of time and, it is considered that, due to the deposition of respiratory droplets released through multiple respiratory events, mask matrix becomes wet and secondary atomization of the droplets was promoted to produce aerosol. Contamination of the mask due to wearing for the prolonged period should also be considered. Park reported the result of culturing bacteria and fungi from outer and inner layers of the masks wore by 109 Japanese people, and it was found that the mean colony counts were 13.4-times higher on the face-side of masks (83). To sum up, the effectiveness of masks, especially universal masking, seems to be limited based on the evidence described above. Regarding this point, the effectiveness of mask rules in schools has not been proven as well. For example, upon comparison of two cities, it was found that recommendation of face mask use in schools for pupils aged 10–12 didn't lower the number of COVID-19 infections (84). Similar results were also reported in school settings in various countries such as Norway (85), U.K (86), and Spain (87).

## Mask rules (mandate): Review of side effects

Further, we should more deeply consider side effects of universal masking for children. Watanabe previously alerted mask addiction (88). Although wearing of masks makes it more difficult to read emotions and provides a temporary sense of security, the continued wearing of masks may diminish this sense of security, leading to a risk of worsening social anxiety.

Not only mental but also physical side effects of masks were well studied. Prolonged mask use is reported to cause headache and impaired cognition (89). Koseoglu et al. also reported increases in dyspnea, itching, ear pain, and headache induction (90). Ou et al. reported negative impact on the ventilation function of exercise with mask on in young healthy subjects (91). It is also important point that the weight of the surgical mask increased during the exercise (92). This means that masks were wet by evaporation of sweat. As described in the previous section, we should consider the accumulation of contaminated droplets for long periods and their release as aerosols. Children's modified Borgi score, an indicator of breath shortness, was significantly increased by the simple exercise with surgical mask compared to not wearing a mask (93).

Difficultly in recognition of emotion is also an important issue when considering universal masking for children. Ruba and Pollak reported aged 7–13 children have significant difficultly in reading emotion (94). In particular, mask inhibited accurate reading of fear emotion <25% (median value). Gori et al. also reported that masks inhibited reading of emotion of toddlers and children (95). Studies reported by Grahlow et al. was like this, and face masks inhibited all kinds of emotion from the face (96). When does emotional development reach adult levels? There are several scientific reports on this issue. Cohen et al. showed that cognitive abilities in emotional situations may be developing even in teenagers and young adults (97). Research on emotional understanding and prosocial behavior have been actively conducted in Japan, and some Japanese papers have been published. For example, Toda reported a significant correlation between emotional cognition and prosocial behavior in young children (98).

## Discussion

Health policies during the COVID pandemic has changed our daily life especially in children in Japan. Children were encouraged to comply with “new way of life” which requires “stay at home”, “mokusyoku”, and wearing a mask all day long. This review summarized the scientific research related to these health policies.

First, I reviewed lockdown and “Stay at home” campaign. The relationship between COVID-19 and obesity in childhood was well reviewed (99). We should have to critically reflect on this health policy has caused changes in eating and exercise habits that increased obesity. The importance of nutritional education to prevent obesity is well studied (100). Dietary intervention programs to prevent body weight gain have also been developed (101). It will be important in the future to use these programs to develop health policies to prevent obesity during pandemic.

Second, I reviewed “mokusyoku rule” and food education and simple school lunch. Close contact is exactly risk factor of COVID infection (102) but for example, hand hygiene could reduce infection (103). In Japan, in-depth research has revealed the beneficial effects of food education and conversation during meals. Nutrients which are effective in preventing infectious diseases are also well studied. Health policies should be developed so that children can take enough these nutrients.

Third, I reviewed the mask rule. There was no mask obligation with penalties in Japan. However, mask wearing was strongly demanded. Over estimation of the effectiveness of mask might be impeding science-based health policy making and infection control. Psychological and physical adverse effect of prolonged mask wearing was also reviewed. As Thomson pointed out, any negative impact on mental or emotional wellbeing experienced by children who are required to wear masks may vary according to age and ability factors and which may yet be established, may be inconsistent with the WHO constitution (104). In the future, it will be necessary to proactively take less burdensome and less legally complex measures, such as adequate hand washing and ventilation.

Vaccination might have one of the key roles of the public health. The efficacy and safety of vaccination for children have been also well studied (105). However, the amount of spike protein synthesized in the body after vaccination has only been measured in adults and there is a discrepancy between the report of Ogata et al. which is in the pico-order (106), and the Cognetti and Miller in the micro-order (107). We should carefully consider the risk and benefits of vaccination and ensure that everyone's judgment is respected. In a recent survey, some parents (8.2%) answered that they intended to vaccinate their children because pediatricians might think less of them if they do not do so (108). This result might suggest that more thorough informed consent is needed. It is a matter of course that misleading media coverage focused only on the benefits or harms of vaccination should be refrained from, as such coverage only contributes to vaccine hesitancy.

As described in Section Introduction, Japan is a unique country that confronts the pandemic without measures with legal binding force, and administrative organs stayed with “recommendation” and avoided orders in most cases. It is reported that most Japanese people think “everyone should be responsible for their health” (109, 110) and should refrain from outside recreation during the pandemic (111). They wear masks (112) and wash their hands (113) voluntarily because they value peer pressure and are afraid of being left out of the community. Television broadcasts, which repeatedly report excessively about facemasks, might also play a part in the formation of the public opinion that it is acceptable to condemn not wearing a mask. Before the pandemic, immoral post for social network service (SNS) by healthcare workers were sometimes came to an issue (114). In the pandemic period, there were not a few posts on SNS by healthcare workers denigrating those who do not want to wear masks and such opinion also might have an influence. Public opinion formed by the accumulation of these factors might influence, sometimes excessively, societal pandemic measures, including in schools.

Health policy should be developed based on multifaced scientific evidence and respect for individual values. Even if the measures have no legal binding force, sometimes measures with greater disadvantages, like those reviewed in this paper, are enforced, especially for children. It is important to regularly receive feedback from schools and review measures from multiple perspectives, including not only the opinion of public health experts but also experts in nutrition science, food education, psychology, and of course, children's opinion and rights.

## Data availability statement

The original contributions presented in the study are included in the article/supplementary material, further inquiries can be directed to the corresponding author.

## Author contributions

NS reviewed the literature and drafted the perspective.

## Conflict of interest

Author NS is also employed by the company Nissin Foods Holdings. The company was not involved in the study design, collection, analysis, interpretation of data, the writing of this article or the decision to submit it for publication.

## Publisher's note

All claims expressed in this article are solely those of the authors and do not necessarily represent those of their affiliated organizations, or those of the publisher, the editors and the reviewers. Any product that may be evaluated in this article, or claim that may be made by its manufacturer, is not guaranteed or endorsed by the publisher.
